# Immune Subversion by *Mycobacterium tuberculosis* through CCR5 Mediated Signaling: Involvement of IL-10

**DOI:** 10.1371/journal.pone.0092477

**Published:** 2014-04-02

**Authors:** Shibali Das, Sayantan Banerjee, Saikat Majumder, Bidisha Paul Chowdhury, Avranil Goswami, Kuntal Halder, Urmita Chakraborty, Nishith K. Pal, Subrata Majumdar

**Affiliations:** 1 Division of Molecular Medicine, Bose Institute, Kolkata, India; 2 Department of Microbiology, Institute of Post Graduate Medical Education and Research, Kolkata, India; 3 Department of Microbiology, Malda Medical College, Malda, India; University of KwaZulu-Natal, South Africa

## Abstract

Tuberculosis is characterized by severe immunosuppression of the host macrophages, resulting in the loss of the host protective immune responses. During *Mycobacterium tuberculosis* infection, the pathogen modulates C-C Chemokine Receptor 5 (CCR5) to enhance IL-10 production, indicating the possible involvement of CCR5 in regulation of the host immune response. Here, we found that *Mycobacterium* infection significantly increased CCR5 expression in macrophages there by facilitating the activation of its downstream signaling. These events culminated in up-regulation of the immunosuppressive cytokine IL-10 production, which was further associated with the down-regulation of macrophage MHC-II expression along with the up-regulation of CCR5 expression via engagement of STAT-3 in a positive feedback loop. Treatment of macrophages with CCR5 specific siRNA abrogated the IL-10 production and restored MHCII expression. While, *in vivo* CCR5 silencing was also effective for the restoration of host immune responses against tuberculosis. This study demonstrated that CCR5 played a very critical role for the immune subversion mechanism employed by the pathogen.

## Introduction

Tuberculosis is a severe chronic bacterial infection caused by the pathogen, *Mycobacterium tuberculosis*, which claims the life of nearly 1.8 million people each year [1]. The pathogen resides within the host macrophages and modulates their pro-inflammatory function [2]. *M. tuberculosis*-infected macrophages actively suppress the host-protective immune responses by secreting high levels of the immunosuppressive cytokine IL-10 [3] and by reducing the expressions of MHC class-II and co-stimulatory molecules [4]–[5]. Thus, the bacilli adopt various immune-evasion strategies that prevent the resolution of its infection.

CCR5 is expressed on a number of hematopoietic and non hematopoietic cells [6]. Ligands for this receptor include MIP-1α, MIP-1β, RANTES and HIV gp120. CCR5 serves as a major co-receptor for HIV [7]. Therefore, targeting CCR5 may acts as an important therapeutic tool against this disease. In murine models of *Cryptococcus neoformans* and *Toxoplasma gondii* infection, CCR5 influences protective immunity [8]–[9]. In contrast, CCR5 plays an antagonistic role against the host during *Leishmania major*, *Paracoccidioides brasiliensis* and HIV infection [10]–[13]. However, the mechanism of CCR5 induction in *Mycobacterium tuberculosis* infected macrophages is yet to be explored.

In the present study, we have demonstrated the mechanism of enhanced CCR5 expression during *Mycobacterium tuberculosis H37Rv* infection. We also observed that infected macrophages secreted high level of anti inflammatory cytokine, particularly IL-10 in CCR5 dependent signaling pathway, along with the down-regulation of classical proinflammatory cytokines. This elevated IL-10 production was responsible for the attenuated MHC-II expression in the infected macrophages. However, CCR5 silencing significantly abrogated the IL-10 mediated immunosuppression generated by the pathogen while inducing higher level of MHC-II expression in infected macrophages. Our *in vivo* experiments strongly suggested that CCR5 played a pivotal role in the regulation of immunity during disease progression. Thus our study might provide crucial cues in understanding the strategy adopted by *Mycobacterium* species to subvert the host immune response in order to progress this dreaded disease.

## Materials and Methods

### Ethics Statement

This study was carried out in strict accordance with the recommendations in the Guide for the Care and Use of Laboratory Animals of the National Institutes of Health. All experimental animal protocols received prior approval from the Institutional Animal Ethical Committee (Bose Institute, Registration Number: 95/99/CPCSEA).

### Reagents and Chemicals

RPMI-1640 medium, penicillin and streptomycin, PD098059 (ERK inhibitor), PP2 (LYN inhibitor), Brefeldin A (Golgi blocker) and TRI Reagent were from Sigma (St Louis, MO, USA). Fetal calf serum (FCS) was purchased from Gibco BRL (Grand Island, NY, USA) and ELISA Assay Kit of mouse IL-10, IL-12, TNF-α, IFN-γ, MIP-1α, MIP-1β and Rantes were from BD and TGF-β was from eBioscience. dNTPs, RevertAidTM M-MuLV Reverse Transcriptase, oligo dT, RNase inhibitor and other chemicals required for cDNA synthesis were from Fermentas (USA). Anti-phospho-H3 and Anti-acetyl-H3 Abs were obtained from Abcam and chromatin immunoprecipitation (ChIP) assay kits were purchased from Millipore (Bedford, MA, USA). GAPDH, phosphorylated and dephosphorylated form of Lyn and ERK-1/2 antibodies, CCR5 antibody were obtained from Santa Cruz Biotechnology (San Jose, CA, USA).

### Animals

8 to 10-wk old female C57BL/6 mice were purchased from the National Centre for Laboratory Animal Sciences, India. All mice were maintained in specific pathogen-free condition. In vivo work was performed in IPGME&R, Kolkata with the help of Dr. Nishith K Pal.

### Bacterial Strain

*Mycobacterium tuberculosis H37Rv* (ATCC 25177) were grown in Middlebrook 7H9 medium (BD Difco, NJ, USA) with 0.02% glycerol, 0.05% Tween 80 and 10% albumin-dextrose complex enrichment (BD Difco, NJ, USA) in shaker flasks. Bacteria were harvested in the mid-log growth phase by centrifugation at 2,500 g for 15 min. The bacteria were then washed twice using the centrifugal washing method and suspended in saline at the desired concentration.

### Preparation of CCR5 Specific Small Interfering RNA and CCR5 Specific shRNA

We synthesized CCR5 specific small interfering RNA (siRNA) using the Silencer siRNA Construction kit (Ambion) [14]. GAPDH siRNA was used for negative control. For sustained gene silencing effect in vivo, short hairpin oligos (shRNA∼50 bases) were synthesized with the same sense and antisense sequences separated by a nine base loop sequences in the middle and a terminator sequence (five to six Ts) at the 3′-end and inserted in the polycloning site of pSilencer 1.0 U6 (mouse) plasmid vector having mouse U6 promoter (Ambion Inc.).

### *In vitro* Transfection of siRNA and Infection of Bone Marrow Derived Macrophages

Macrophages were grown from murine bone marrow precursors and cultured for 5 days using methods described elsewhere [15]. Adherent macrophages were transfected with CCR5 siRNA, STAT-3 siRNA (Santacruz) and GAPDH siRNA (100 nM), using transfection reagent Oligofectamine (Invitrogen) as per manufacturer’s instructions [14]. After 24 h of incubation the macrophages were infected with *Mycobacterium tuberculosis H37Rv* (mid log phase) at a ratio of 1∶10 (macrophage: *Mycobacteria*).

### *In vivo* Studies

For in vivo studies C57BL/6 mice were divided into following groups based on the regime of treatment: (1) control mice (administered phosphate-buffered saline); (2) *M. tuberculosis*–infected mice. The mice were infected with a volume of bacterial suspension, and exposure time was calibrated to deliver∼100 CFU per animal. To determine the infection dose, lung inoculum was verified by agar plating 24 h after infection as described elsewhere [16]. (3) The mice were treated with either CCR5 shRNA plasmids or with Control ShRNA plasmid (pSilencer 1.0) by hydrodynamic tail vein injection 3 days prior to infection. After 28 days of infection, mice were sacrificed and their organs were removed aseptically.

### Flow Cytometry

Macrophages were stained with phyco-erythrin (PE)–labeled anti-CCR5 and anti-MHCII antibodies (Santa Cruz Biotech). Cells were analyzed using a FACS Verse flow cytometer (Becton Dickinson).

### Cytokine Determination

Cytokines were measured from infected cell supernatants with mouse IL-10, TGF- β, IFN-γ, IL-12, TNF- α, MIP-1α, MIP-1β and Rantes ELISA Sets. In vivo cytokines were measured from lung homogenate extracted by centrifuging homogenized lung tissue to create a tissue-free supernatant as described elsewhere [17].

### Preparation of Cell Lysate and Immunoblot Analysis

Cell lysates from infected macrophages were prepared as described elsewhere [18]. Equal amounts of protein (50 ug) were subjected to 10% sodium dodecyl sulfate polyacrylamide gel electrophoresis, and immunoblotting was performed as described elsewhere [19].

### Isolation of RNA and Semi Quantitative Polymerase Chain Reaction

Total RNA extracted from macrophages and lungs of respective animals (TRI reagent; Sigma). For cDNA synthesis, 1 μg of total RNA from each sample was reverse-transcribed using Revert Aid M-MuLV Reverse Transcriptase (Fermentas). cDNA from each sample was amplified with 0.5 unit Taq DNA polymerase (Fermentas) in 50 μl reaction volume under the following conditions: initial activation step (2min at 95°C) and cycling step (denaturation for 30 s at 94°C, annealing for 30 s at 58°C, and extension for 1 min at 72°C for 35 cycles), using Perkin Elmer Gen Amp PCR system 2400. Sequences of the PCR primers are listed in Table 1. PCR amplified products were subsequently size fractioned on 1.5% agarose gel, stained with ethidium bromide and visualized under UV-light.

**Table 1:** List of Primers.

IFN-γ	Forward 5′-GGATATCTGGAGGAACTGGC-3′
	Reverse 5′-CGACT CCTTTTCCGCTTCCT-3′
IL-10	Forward 5′-CGGGAAGACAATAACTG-3′
	Reverse 5′-CATTTCCGATAAGGCTTGG-3′
TGF-β	Forward 5′-GGATACCAACTATTGCTTCAGCTCC-3′
	Reverse 5′-AGGCTCCAAATATAGG GGCAGGGTC-3′
IL12p40	Forward 5′-CAACATCAAGAG CAGTAGCAG-3′
	Reverse 5′TACTCCCAGCTGACCTCCAC-3′
TNF-	Forward 5′-GGCAGGTCTACTTT GGAGTCATTGC-3′
	Reverse 5′-ACATTCGAGGCTCCAGTGAATTCGG-3′
MIP-1α	Forward 5′-TCATCGTTGACTATTTTGAAACCAG-3′
	Reverse 5′-GCCGGTTTCTCTTAGTCAGGAA-3′
MIP-1β	Forward 5′-TGCTCGTGGCTGCCTTCT-3′
	Reverse 5′- CTGCCGGGAGGTGTAAGAGA-3′
RANTES	Forward 5′-CCCTCTGCACCCCCGTACCT-3′
	Reverse 5′-CCATTTTCCCAGGACCGAGT-3′
CCR5	Forward: 5′-AATAATTGCAGTAGCTCTAACAGG-3′
	Reverse: 5′-TTGAGTCCGTGTCACAAGCCC-3′
GAPDH	Forward 5′-CAAGGCTG TGGGCAAGGTCA-3′
	Reverse 5′-AGGTGGAAGAGTGGGAGTTGCTG-3′
IL-10 Promoter	Forward 5′-TCATGCTGGGATCTGAGCTTCT-3′
	Reverse 5′-CGGAAGTCACCTTAGCACTCAGT-3′
CCR5 Promoter	Forward 5′-TGTGGGCTTTTGACTAGATGA-3′
	Reverse 5′-TAGGGGAACGGATGTCTCAG-3′
CCR5 siRNA	Forward 5′-AACAGGTCAGAGATGGCCAGGCCTGTCTC-3′
	Reverse 5′-AACCTGGCCATCTCTGACCTGCCTGTCTC-3′

### Chromatin Immuno Precipitation (CHIP) Assay

CHIP assays were conducted using the CHIP Assay kit following the manufacturers Protocol (Millipore) as described elsewhere [20]. Briefly, Cells were collected after infection for indicated periods and nuclear extraction was performed. Protein-DNA complexes were immunoprecipitated with 5 ug of Ab (Phospho-H3, Acetyl-H3, STAT-3) overnight at 4°C. Ab-protein-DNA complexes were then captured by protein A-agarose for 1 h at 4°C. After washing beads with different buffers, the protein/DNA complexes were eluted using 1% SDS, 0.1 M NaHCO3 buffer and disrupted by heating at 65°C for 4 h. DNA was then extracted using phenol/chloroform extraction and ethanol precipitation. PCR was conducted using promoter specific primers (Table 1; PCR condition: 94°C, 15 s; 56°C, 30 s; 72°C, 1 min, 35cycles). PCR amplified product was subsequently size fractioned on 1.5% agarose gel, stained with ethidium bromide and visualized under UV-light.

### Determination of Colony Forming Unit (CFU) Counts

Lung and spleen of different group of mice were lysed at 28 days post-infection and respective *M. tuberculosis* containing suspensions were repeatedly passed through a 30-gauge needle to obtain predominantly single cell suspension. This lysate was serially diluted and plated on Middlebrook 7H10 with Oleic acid-ADC in triplicate. Colony forming units (CFU) were counted after 21 days of incubation at 37°C. Data are expressed as log_10_CFU per organ as the Mean ± standard deviation.

### Statistical Analysis

For in vivo experiments, a minimum of five mice were used per group. The experiments were performed at least three times and the data presented as means ± SD. Student’s t-test or one-way ANOVA was employed to assess the significance of the differences between the mean values of the control and experimental groups. A P value <0.05 was considered significant and <0.001 was considered highly significant.

## Results

### 1. *Mycobacterium tuberculosis H37Rv* Induces the Enhancement of CCR5 Expression in Host Macrophages

**Figure 1:**
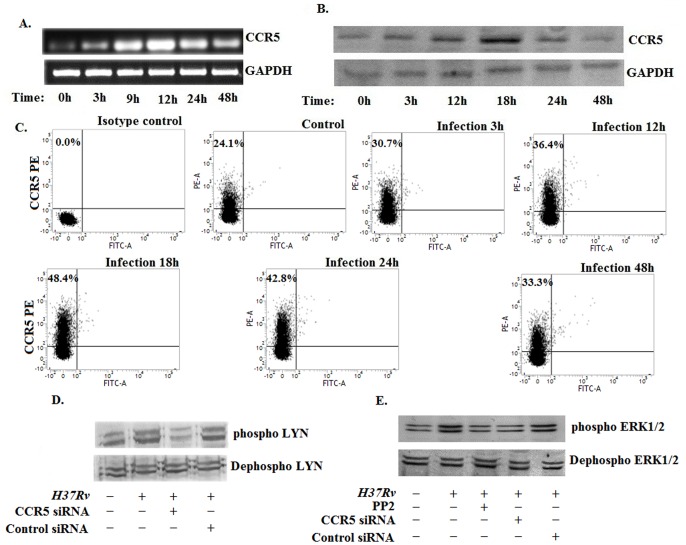
*Mycobacterium tuberculosis H37Rv* induces the enhancement of CCR5 expression in host macrophages. Bone marrow derived macrophages (2×10^6^) were cultured and then infected with *Mycobacterium tuberculosis H37Rv* [Multiplicity of Infection (MOI) = 1∶10] for different time points. Changes in messenger RNA (mRNA) expression of CCR5 and GAPDH were determined by semi quantitative RT-PCR (A). In a separate set, the infected macrophages were lysed and subjected to Western blot with anti-CCR5 antibody as described in Materials and Methods (B). Infected macrophages were analyzed by flow cytometry for CCR5 (PE) expression as described in material method (C). In a separate set, macrophages were treated with either CCR5 siRNA or Control siRNA or Lyn inhibitor PP2 and then infected as described above and cell lysates were subjected to western blot with either anti-Lyn antibody (D) or anti-ERK antibody (E). Data represented here are from one of three independent experiments, all of which yielded similar results.

The previous studies suggested that the CC chemokine receptor 5 (CCR5) was involved in the disease progression process and knockdown of this receptor lead to the resolvation of the disease [21]. But the detailed study on the involvement of CCR5 during disease progression is not clear till date. Therefore, we investigated whether *Mycobacterium tuberculosis H37Rv* infection augmented the CCR5 expression in murine bone marrow derived macrophages. Semi- Quantitative RT PCR studies revealed a gradual increase in CCR5 expression in infected macrophages compared to the uninfected control macrophages (Figure 1A). We found that the CCR5 expression was a time dependent phenomenon, started within 3 h post infection and peaked around 12 h post infection followed by a moderate decline at the later time points of infection. In addition, we investigated the CCR5 expression in *H37Rv* infected macrophages by western blot and FACS (Figure 1B and 1C). These studies exhibited similar pattern of CCR5 expression as observed in Figure 1A. Therefore, these findings clearly indicated that *Mycobacterium tuberculosis H37Rv* induced a gradual increase in CCR5 expression during host pathogen interaction.

To understand the mechanistic insight, we studied the CCR5 downstream signaling. It was observed that Lyn, a src kinase was phosphorylated and activated during *Mycobacterium tuberculosis H37Rv* infection (Figure 1D.). In contrast, Lyn phosphorylation was significantly abrogated in infected macrophages pretreated with CCR5 specific siRNA. It was reported that Lyn kinase induced the ERK1/2 phosphorylation in HIV infection [22]. We observed that *Mycobacterium tuberculosis H37Rv* infection also induced ERK1/2 phosphorylation (Figure 1E). In contrast when we pretreated the macrophages with either CCR5 specific siRNA or with the pharmacological inhibitor of Lyn kinase (PP2), the ERK1/2 phosphorylation was significantly abrogated. Here we suggest that MTB infection enhanced the CCR5 expression thereby activating its downstream signaling.

### 2. *Mycobacterium tuberculosis H37Rv* Infection Disrupts the Pro-inflammatory Function of CCR5 in Macrophages to Produce High Level of IL-10

**Figure 2:**
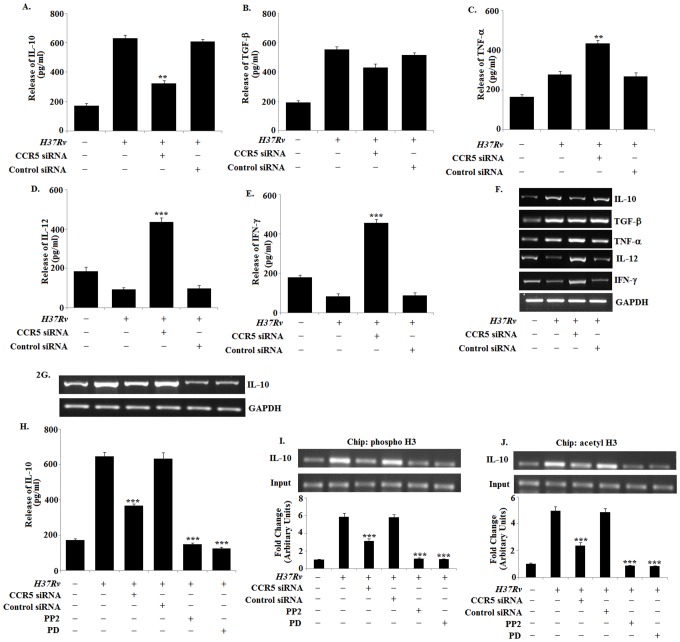
*Mycobacterium tuberculosis H37Rv* infection disrupts the proinflammatory function of CCR5 in macrophages to produce high level of IL-10. Bone marrow derived macrophages (2×10^6^ cells/ml) were either treated with CCR5 siRNA or Control siRNA followed by *Mycobacterium tuberculosis H37Rv* (MOI = 1∶10) infection for 24 h and assayed for the levels of IL-10 (A), TGF-β (B), TNF-α (C), IL-12 (D) and IFN-γ (E) in the culture supernatant by ELISA as described in Methods. ELISA data are expressed as means ± standard deviations of values from triplicate experiments that yielded similar observations. ***P<.001 and **P<.05 compared to that of the infected macrophages. In a separate set of experiment, siRNA pre treated macrophages were infected with *H37Rv* for 3 h. The extracellular bacteria were removed from the culture plate and the macrophages were incubated for another 3 hrs. RNA was isolated and semi quantitative RT-PCR analyses for TNF-α, IL-12, IFN-γ, IL-10, TGF-β and GAPDH were done. Data represented here are from one of three independent experiments, all of which yielded similar results (F). In separate experimental sets, Murine macrophages were pretreated with either control siRNA, CCR5-specific siRNA for 24 h or with Lyn inhibitor PP2, ERK inhibitor PD for 2 h, washed and then infected with *Mycobacterium* tuberculosis and changes in messenger RNA (mRNA) expression of IL-10 and GAPDH were determined by semi quantitative RT-PCR (G). Murine macrophages were transfected with siRNA and infected as mentioned above for 24 h and assayed for the levels of IL-10 (H) in the culture supernatant by ELISA as described in Methods. ELISA data are expressed as means standard deviations of values from triplicate experiments that yielded similar observations. ***P<.001 and **P<.05 compared to that of the control siRNA treated infected macrophages. Murine macrophages (1×10^6^cells/ml) were treated as described previously and then subsequently followed by *Mycobacterium* tuberculosis infection for 45 min. After 45 min of incubation, ChIP assays were conducted as described in Materials and Methods. Immunoprecipitations were performed using Abs specific to phosphorylated H3 (IP phospho-H3) (I) or acetylated H3 (IP acetyl-H3) (J), and conventional RT-PCR was performed using primers specific to the IL-10 promoter. Data represented here are from one of three independent experiments, all of which yielded similar results.

Augmentation of CCR5 expression is accompanied with the increase in pro-inflammatory cytokine and chemokine production in macrophages [8]. Therefore, we investigated whether enhancement of CCR5 expression in macrophages during *H37Rv* infection was associated with increased pro-inflammatory cytokine and chemokine production (Figure S1). We observed significant increase in the production of IL-10 (Figure 2A and 2F) and TGF-β (Figure 2B and 2F) and slightly higher level of TNF-α (Figure 2C and 2F) in the infected macrophages as compared with the uninfected control macrophages. However, blocking of CCR5 by CCR5 specific siRNA significantly down-regulated the IL-10 expression and TGF-β expression to a moderate level. Interestingly TNF-α expression was enhanced in the CCR5 knockdown infected macrophages. On the contrary we observed decreased level of IL-12 (Figure 2D and 2F) and IFN-γ (Figure 2E and 2F) in the infected macrophages which were comparable to the control macrophages. Blocking of CCR5 significantly up-regulated both IL-12 and IFN-γ level in infected macrophages. Therefore, these findings clearly indicated for the first time that *H37Rv* infected macrophages modulated the CCR5 function to produce immunosuppressive cytokine IL-10 instead of inducing pro-inflammatory cytokines during the course of *Mycobacterium* infection.

To find out the reason, we studied the signaling mechanism involved in the production of IL-10 by exploiting CCR5 during the disease progression. Earlier we have shown above that the CCR5 downstream signaling activated the kinase Lyn and ERK in infected macrophages (Figure 1). Specific inhibition of either CCR5 itself or its downstream mediators Lyn (PP2) or ERK (PD) significantly ablated IL-10 expression in infected macrophages (Figure 2G and 2 H). We also observed the involvement of other receptor in the production of IL-10 during tuberculosis (Figure S2).

To explore the mechanism behind the regulation of IL-10 we examined the core histone modification at the IL-10 promoter by ChIP assays. We observed that, *Mycobacterium tuberculosis H37Rv* infection was associated with significantly augmented histone phosphorylation and acetylation at the IL-10 locus (Figure 2I and 2J) whereas CCR5 silencing or inhibition of Lyn or ERK1/2 resulted in drastic reduction of both histone phosphorylation and acetylation at the same locus. These results demonstrated that CCR5, along with Lyn and ERK-1/2, is critical *for Mycobacterium tuberculosis H37Rv* elicited responses. Taken together, these findings identify a novel pathway involving CCR5-mediated activation of the Src kinase Lyn and the MAP kinase ERK-1/2 in IL-10 production following macrophage engagement by *Mycobacterium tuberculosis H37Rv*.

### 3. Involvement of CCR5 Dependent IL-10 Activation in the Down-regulation of MHC-II Expression in *H37Rv* Infected Macrophage

**Figure 3:**
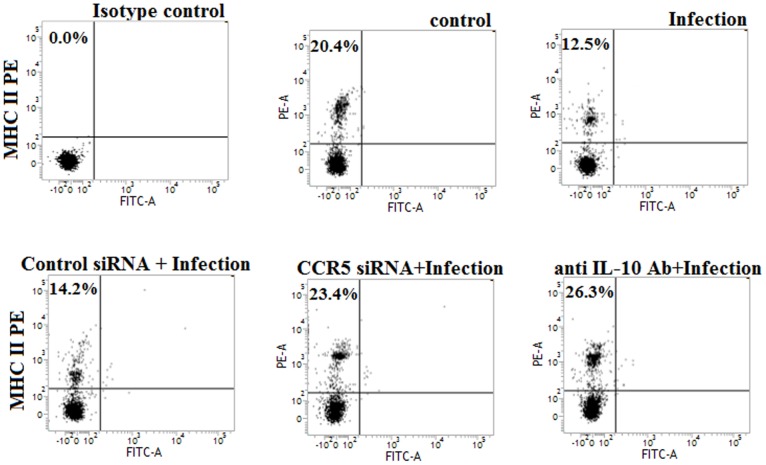
The down-regulation of MHC-II expression in *H37Rv* infected macrophages was due to the CCR5 dependent IL-10 activation. Murine macrophages (2×10^6^cells/ml) were stimulated with IFN-γ (2 ng/ml) and then pretreated with either control siRNA or CCR5-specific siRNA for 24 h and 10 ug/ml anti IL-10 Ab for 1 h followed by *Mycobacterium* tuberculosis infection for 24 h. Infected macrophages were analyzed by flow cytometry for MHC-II (PE) expression as described in material method (A). Data represented here are from one of three independent experiments, all of which yielded similar results.

IL-10 plays a crucial role for the down-regulation of MHC-II expression in antigen presenting cells during the course of various infections [23]–[24]. Here we investigated whether IL-10 was responsible for the regulation of MHC-II expression in macrophages during *H37Rv* infection. Bone marrow derived macrophages express very low level of MHC-II under normal (unstimulated) condition [25]. Therefore, we pre-stimulated the macrophages with IFN-γ in order to enhance the MHC-II expression. We observed significant abrogation of MHC-II expression in *H37Rv* infected macrophages compared to the IFN-γ stimulated uninfected control macrophages (Figure 3A). Interestingly, IL-10 neutralizing antibody pre-treatment completely restored the MHC-II expression in IFN-γ stimulated *H37Rv* infected macrophages (Figure 3A). Furthermore, we pre-treated the macrophages with CCR5 siRNA to investigate whether CCR5 derived IL-10 was involved in the regulation of MHC-II expression in IFN-γ stimulated *H37Rv* infected macrophages. Interestingly, CCR5 siRNA pre-treatment resulted in a significant restoration of MHC-II expression in IFN-γ stimulated *H37Rv* infected macrophages compared to the control siRNA treated IFN-γ stimulated infected macrophages. Therefore, these findings clearly indicated that CCR5 induced IL-10 production was responsible for the attenuation of MHC-II expression in macrophages during *H37Rv* infection.

### 4. IL-10 Augments the CCR5 Expression in *H37Rv* Infected Macrophages via Involving STAT3

**Figure 4:**
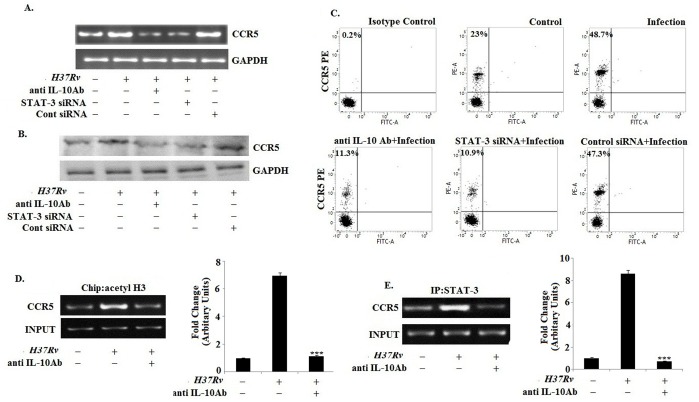
IL-10 augments the CCR5 expression in *H37Rv* infected macrophages via involving STAT3. Bone marrow derived macrophages (2×10^6^cells/ml) were pretreated with either anti IL-10 Ab (10 ug/ml) or with control siRNA and STAT3-specific siRNA and then infected with *Mycobacterium tuberculosis H37Rv* (MOI = 1∶10). Changes in messenger RNA (mRNA) expression of CCR5 and GAPDH were determined by semi quantitative RT-PCR (A). In a separate set, the pretreated and infected macrophages were lysed and subjected to Western blot with anti-CCR5 antibody as described in Materials and Methods (B). Infected macrophages were analyzed by flow cytometry for CCR5 (PE) expression as described in figure legend 1 (C). Data represented here are from one of three independent experiments, all of which yielded similar results. Murine macrophages (1×10^6^cells/ml) were treated with anti IL-10 Ab for 1 h and then subsequently followed by *Mycobacterium* tuberculosis infection for 45 min. After 45 min of incubation, ChIP assays were conducted as described in Materials and Methods. Immunoprecipitations were performed using Abs specific to acetylated H3 (IP acetyl-H3) (D) or STAT-3 (E), and conventional RT-PCR was performed using primers specific to the CCR5 promoter. Data represented here are from one of three independent experiments, all of which yielded similar results.

Recent studies provide ample evidences that recombinant IL-10 is capable of inducing CCR5 in human monocyte [26] and in a macrophage like cell line HL-60 [27]. Therefore, we intended to investigate whether IL-10 played a similar role in the regulation of CCR5 expression in macrophages during *H37Rv* infection. IL-10 neutralizing antibody significantly abrogated the CCR5 expression in macrophages during the course of *H37Rv* infection (Figure 4A, 4B and 4C). To further prove the role of cytokine in the up-regulation of CCR5 during infection, we studied the expression of CCR5 in the presence of Brefeldin A (Figure S3). Moreover, IL-10 executes most of its immunomodulatory effects through the activation of the transcription factor STAT3. Interestingly, pre-treatment of the macrophages with a STAT3 specific siRNA completely abrogated the IL-10 induced enhancement of CCR5 expression in *H37Rv* infected macrophages.

To understand the mechanism behind the regulation of CCR5 expression, we examined the core histone modifications at the CCR5 promoter region in *H37Rv* infected macrophages by ChIP assay. *Mycobacterium tuberculosis H37Rv* infection in macrophages was accompanied with high amount of acetylated histone at the CCR5 locus in macrophages compared to the uninfected control macrophages (Figure 4D). We observed STAT-3 binding at the CCR5 promoter region in *H37Rv* infected macrophages (Figure 4E). However, pre-treatment with the IL-10 neutralizing antibody prior to *Mycobacterium tuberculosis H37Rv* infection exhibited a sharp decline in STAT-3 binding at CCR5 locus in infected macrophages. Therefore, these findings indicated that the enhanced CCR5 expression in *H37Rv* infected macrophages was associated with the IL-10 mediated STAT3 binding at the CCR5 promoter.

### 5. Effect of CCR5 on the Survival of *Mycobacterium tuberculosis*

**Figure 5:**
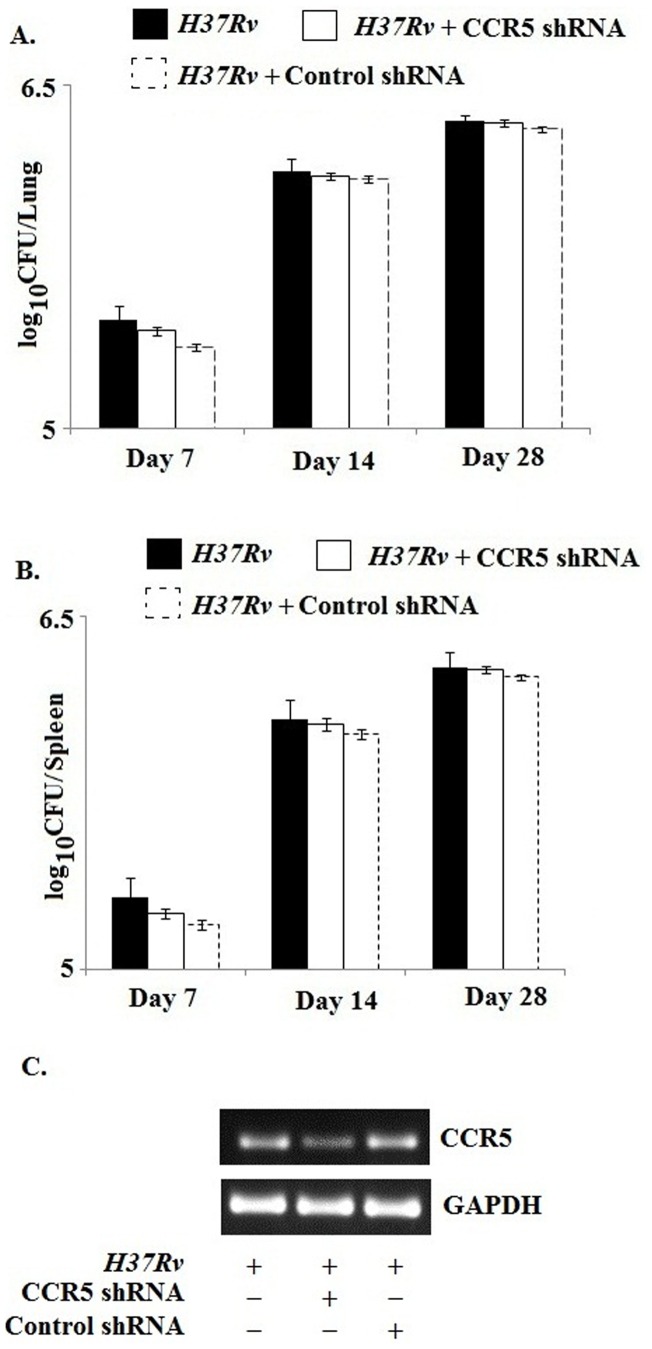
Effect of CCR5 on the survival of *Mycobacterium tuberculosis.* C57BL/6 mice were transfected with CCR5 shRNA or control shRNA as described in materials and methods section prior to *Mycobacterium* infection. After 28 days of infection, the lungs and spleens were lysed. The respective lysates were serially diluted and plated on Middle brook 7 H10 with Oleic acid-ADC in triplicate. Data are represented as log_10_CFU/organ as mean ± SD. In a separate set, the transfected and infected mice were sacrificed and then the CCR5 expression in infected macrophages were analysed by semi quantitative RT-PCR (C) to validate the specific activity of shRNA mediated knockdown. Data represented here are from one of three independent experiments, all of which yielded similar results.

Our previous findings support that during *H37Rv* infection the CCR5 downstream signaling was activated which in turn augmented the anti inflammatory cytokines at the site of infection. Therefore, we aimed to study whether this receptor mediated signaling have any effect on the growth and survival of the bacteria within the host. Surprisingly we observed no significant change in the Colony Forming Unit (CFU) count in both the lung and spleen of CCR5 shRNA pre treated infected mice as compared with the only infected mice (Figure 5A and 5B). Therefore this finding indicated that CCR5 and its downstream signaling were employed by the pathogen for establishing the immuno suppression within the host without effecting its own survival.

### 6. CCR5 Silencing Enhances Pro Inflammatory Cytokine Production and MHC-II Expression in *H37Rv* Infected C57BL/6 Mice

**Figure 6:**
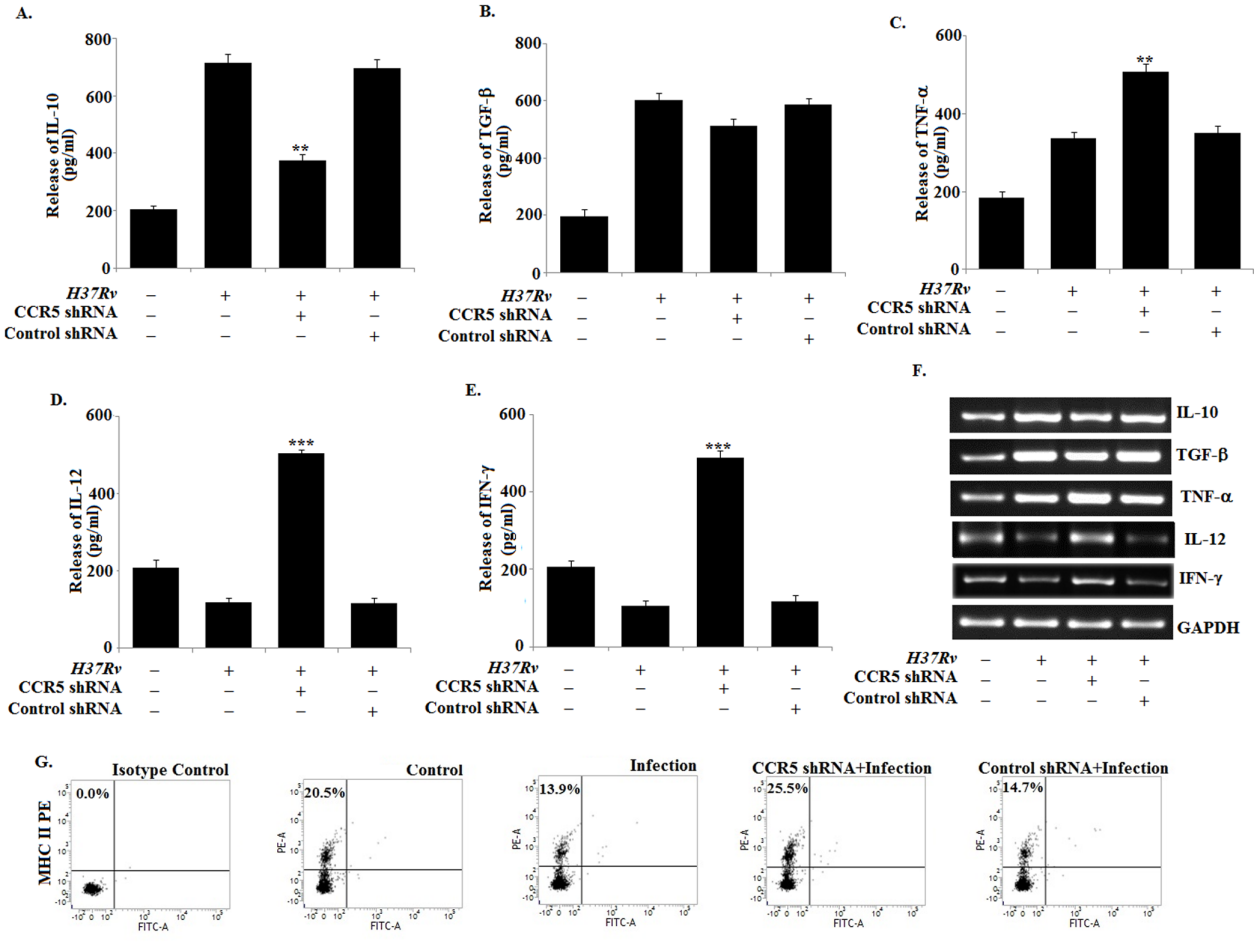
CCR5 silencing enhances pro inflammatory cytokine production and MHC-II expression in *H37Rv* infected C57BL/6 mice. C57BL/6 mice were transfected with CCR5 shRNA or control shRNA as described in materials and methods section prior to *Mycobacterium* infection. After 28 days of infection, the lung homogenates were assayed for the cytokine levels by ELISA as described in Methods (A–E). Data represented as means ± SD for 5 animals per group. ***P<.001 and **P<.05 for the comparison with infected mice. Total lung RNA was extracted and Changes in messenger RNA (mRNA) expression of IL-10, TGF-β, TNF-α, IL-12, IFN-γ and GAPDH were determined by semi quantitative RT-PCR (F). Data represented here are from one of three independent experiments, all of which yielded similar results. In separate experimental set, the lung homogenates were analyzed by flow cytometry for MHC-II (PE) expression as described in figure legend 1(G). Data represented here are from one of three independent experiments, all of which yielded similar results.

To validate our *in vitro* findings, we performed additional studies in C57BL/6 mice to determine the role of CCR5 during *H37Rv* infection *in vivo*. Interestingly, silencing of CCR5 with a CCR5 specific shRNA resulted in a significant attenuation of IL-10 production in the lung homogenate of *H37Rv* infected mice compared to that of the only infected mice (Figure 6A and 6F). Moreover, we observed a significant enhancement in the TNF- α, IL-12 as well as IFN-γ production in the lung homogenate of CCR5 silenced infected mice compared to that of the only infected mice (Figure 6C, 6D, 6E and 6F).

In addition, CCR5 silencing was accompanied with a sharp increase in the MHC-II expression in the lung homogenate of *H37Rv* infected mice (Figure 6G). These findings are clearly indicative of the fact that CCR5 silencing was associated with an increase in pro-inflammatory cytokine production and MHC-II expression in *H37Rv* infected C57BL/6 mice.

## Discussion

In this study, the role of the chemokine receptor, CCR5, was studied in macrophages during *Mycobacterium tuberculosis H37Rv* infection. We observed gradual augmentation of CCR5 expression in *H37Rv* infected murine macrophages (Bone Marrow Derived Macrophages) with the activation of CCR5 downstream signaling mediators (Figure 1).

The chemokine receptor, CCR5, upon binding to its cognate ligand induces the production of pro-inflammatory cytokinesin uninfected control macrophages [28]. Here, we observed significantly higher expressions of MIP-1α, Rantes, MIP-1β (Figure S1) as well as IL-10, TGF-β and moderate level of TNF-α expression in infected macrophages (Figure 2) which was observed by other groups [29]–[30]. In fact, CCR5 silencing led to significant lower production of IL-10 in infected macrophages as compared with control siRNA treated infected macrophages. However, CCR5 silencing also led to enhanced production of TNF- α and other pro inflammatory cytokines such as IL-12 and IFN-γ. Therefore, our results implicated that during infection the CCR5 signaling was modulated by the bacteria in order to subvert the host immune response.

Many other receptors are reported to be involved in the disease progression during tuberculosis. Among them, the most important receptor is TLR2 which is involved in the production of IL-10 during tuberculosis [31]–[32]. Therefore, we studied the involvement of TLR-2 receptor along with the CCR5 in the context of IL-10 production during the course of infection. We blocked the CCR5 and TLR2 receptor by CCR5 specific siRNA and Cobra peptide respectively and evaluated the production of IL-10 during infection (Figure S2). We observed that both the receptors mentioned above, were involved in the regulation of IL-10 production. Therefore, we can assume that during infection, these two receptors downstream signaling may act in a concerted manner to suppress the host immune responses.

IL-10 has a profound role in the down-regulation of MHC-II expression in antigen presenting cells [23]–[24]. Interestingly, inhibition of IL-10 with an IL-10 neutralizing antibody restored the MHC-II expression in *Mycobacterium tuberculosis* infected macrophages (Figure 3). Moreover, we studied whether IL-10 was involved in the regulation of CCR5 expression in macrophages during *Mycobacterium tuberculosis* infection. We observed that the cell free supernatant from the infected macrophages were able to up-regulate the CCR5 expression in uninfected macrophages which was completely abrogated when the cell free supernatant were collected from the Brefeldin A pretreated infected macrophages (Figure S3) where Brefeldin A pretreatment inhibited the cytokine release in the cell free supernatant. Therefore, it is clear that some soluble mediators which were present in the cell free supernatant were responsible for the up-regulation of CCR5 in macrophages. Interestingly, IL-10 neutralizing antibody treatment abrogated the enhanced CCR5 expression in *H37Rv* infected macrophages (Figure 4A, 4B and 4C). Inhibition of the IL-10 down-stream effector, STAT3, exhibited similar effects on CCR5 expression in *H37Rv* infected macrophages. During infection the transcription favorable histone modification was observed in CCR5 promoter region (Figure 4D). Previously, it has been reported that the transcription factors such as CCAAT-Enhancer-Binding Proteins (C/EBPβ) and cAMP Responsive Element Binding protein (CREB) which are crucial for the transcriptional activation of CCR5 gene, found to be severely down regulated during tuberculosis [33]–[35]. Moreover, during infection, transcriptional activation of CCR5 was mostly dependent on STAT3 (Figure 4E). Therefore, IL-10 and its down-stream effector, STAT3, were the essential factors responsible for the enhanced CCR5 expression in macrophages during *H37Rv* infection. This is the first report suggesting a positive feedback loop exists for the dual regulation of CCR5 and IL-10 during the course of infection (Figure S4).

Although CCR5 signaling was involved in the production of anti-inflammatory cytokine but this receptor seemed to have no effect on the survival of the pathogen within the host cells (Figure 5). This was also reported for other receptors which were associated with the entry for the pathogen [21] [36]–[38]. The reason behind this unusual behavior of the pathogen might be the involvement of an array of receptors for the entry of pathogen at a time. Thus, blocking of any particular receptor at a time is not enough to restrict the growth and survival of the pathogen. Therefore, we suggest that CCR5 signaling may improve the overall pathology in terms of the immune-suppression as well as immune-subversion whereas CCR5 alone may not be able to regulate the fate of the pathogen within the host.

To validate our *in vitro* findings, we carried out *in vivo* studies in CCR5 silenced *Mycobacterium tuberculosis* infected mice. Interestingly, CCR5 silencing was associated with a high level of pro-inflammatory cytokine expression i.e. IL-12, TNF-α and IFN-γ along with the restoration of MHC-II expression in the lung of *Mycobacterium tuberculosis* infected mice (Figure 6). IFN-γ increases the expression of MHC-II in alveolar macrophages and activates the macrophages to exert microbicidal functions [39]–[40]. On the other hand, TNFα exhibits host protective function against *Mycobacterium tuberculosis* infection [41]–[43], since anti-TNF-α treatment enhances the susceptibility of the infection [44]. Therefore, CCR5 silencing might have a potential role in the resolution of disease progression which was further associated with a sharp decrease in the IL-10 production. It is known that IL-10 suppresses macrophage and DC functions through the suppression of IL-12, IFN-γ [45]–[46] and MHC II expression during tuberculosis. However, it is likely that in the absence of IL-10, both IL-12 and IFN-γ along with increased MHC-II expression restore the host protective immune responses in CCR5 silenced mice. Therefore, CCR5 silencing plays an important role in the context of host immune response against tuberculosis.

Overall, these findings from both *in vitro* and *in vivo* models of tuberculosis point toward a novel mechanism of CCR5–mediated altered cellular signaling where CCR5 signaling was used by the pathogen as an important strategy to subvert the host immune responses. Moreover, *M. tuberculosis* enhances the CCR5 surface expression in macrophages and renders the macrophage cell lineage more susceptible towards HIV infection [47]. Therefore, we suggest that *Mycobacterium* may employ the above strategies to enhance CCR5 expression which accelerates the disease progression during HIV infection. To our knowledge, this is the first report that unveils the exploitation of CCR5 and its downstream signaling by *Mycobacterium* for establishment of this disease.

## Supporting Information

Figure S1 **Production of chemokines during** ***M. tuberculosis*** **infection.** Bone marrow derived macrophages (2×10^6^ cells/ml) were infected with *Mycobacterium tuberculosis H37Rv* (MOI = 1∶10) for 24 h and assayed for the levels of MIP-1α (A), MIP-1β (B) and Rantes (C) in the culture supernatant by ELISA as described in Methods. ELISA data are expressed as means ± standard deviations of values from triplicate experiments that yielded similar observations. ***P<.001 and **P<.05 compared to that of the uninfected control macrophages. In a separate set of experiment, macrophages were infected with *Mycobacterium tuberculosis H37Rv* for 3 h. The extracellular bacteria were removed from the culture plate and the macrophages were incubated for another 3 hrs. RNA was isolated and semi quantitative RT-PCR analysis for MIP-1α, MIP-1β, Rantes and GAPDH were done (D). Data represented here are from one of three independent experiments, all of which yielded similar results.
(TIF)

Figure S2 **Involvement of TLR-2 and CCR5 in the** ***M. tuberculosis*** **elicited IL-10 production.** Bone marrow derived macrophages (2×10^6^cells/ml) were treated with CCR5 siRNA, control siRNA, TLR-2 inhibitory peptide (Cobra peptide) and control peptide. The macrophages were then infected with *Mycobacterium tuberculosis H37Rv* (MOI = 1∶10) for 24 h and assayed for the levels of IL-10 in the culture supernatant as described above (A). ELISA data are expressed as means ± standard deviations of values from triplicate experiments that yielded similar observations. ***P<.001 and **P<.05 compared to that of the uninfected control macrophages. In a separate set of experiment, macrophages were treated as above and infected with *Mycobacterium tuberculosis H37Rv* for 3 h. The extracellular bacteria were removed from the culture plate and the macrophages were incubated for another 3 hrs. RNA was isolated and semi quantitative RT-PCR analyses for IL-10 and GAPDH were done. Data represented here are from one of three independent experiments, all of which yielded similar results. In a separate set of experiment, macrophages were infected with *Mycobacterium tuberculosis H37Rv* for different time points (B). RNA was isolated and semi quantitative RT-PCR analyses for IL-10 and GAPDH were done. *Mycobacterium tuberculosis H37Rv* (MOI = 1∶10) infected macrophages assayed for the levels of IL-10 in the culture supernatant as described above (A). ELISA data are expressed as means ± standard deviations of values from triplicate experiments that yielded similar observations.
(TIF)

Figure S3 **Involvement of cytokine in the** ***M. tuberculosis*** **elicited CCR5 expression.** Bone marrow derived macrophages (2×10^6^) were cultured. The macrophages were treated with Brefeldin A and then infected with *Mycobacterium tuberculosis H37Rv* (MOI = 1∶10) for 24 hrs. The cell supernatants were mixed with the culture media of uninfected macrophages. Infected macrophages were analyzed by flow cytometry for CCR5 (PE) expression as described in material method (A). In a separate set, the macrophages were treated above and changes in messenger RNA (mRNA) expression of CCR5 and GAPDH were determined by semi quantitative RT-PCR (B). Data represented here are from one of three independent experiments, all of which yielded similar results.
(TIF)

Figure S4 **Schematic representation of the CCR5 signaling pathway during Mycobacterium infection in macrophages.** During early time point of *H37Rv* infection, the CCR5 expression is up-regulated, which in turn activates the Lyn kinase. Phosphorylated Lyn further activates the MAP kinase ERK1/2. This signaling is culminated with the production of IL-10 in the infected macrophages. The IL-10 produced from the infected macrophages recruits STAT-3 to the CCR5 promoter of the infected macrophage and further upregulates the CCR5 expression in the infected macrophages through feedback loop mechanism.
(TIF)
