# Supplementary figures and images for: Immune Subversion by Mycobacterium tuberculosis through CCR5 Mediated Signaling: Involvement of IL-10
Source: PLoS One. 2014 Apr 2;9(4):e92477. doi: 10.1371/journal.pone.0092477 (PMC3973661; doi:10.1371/journal.pone.0092477)

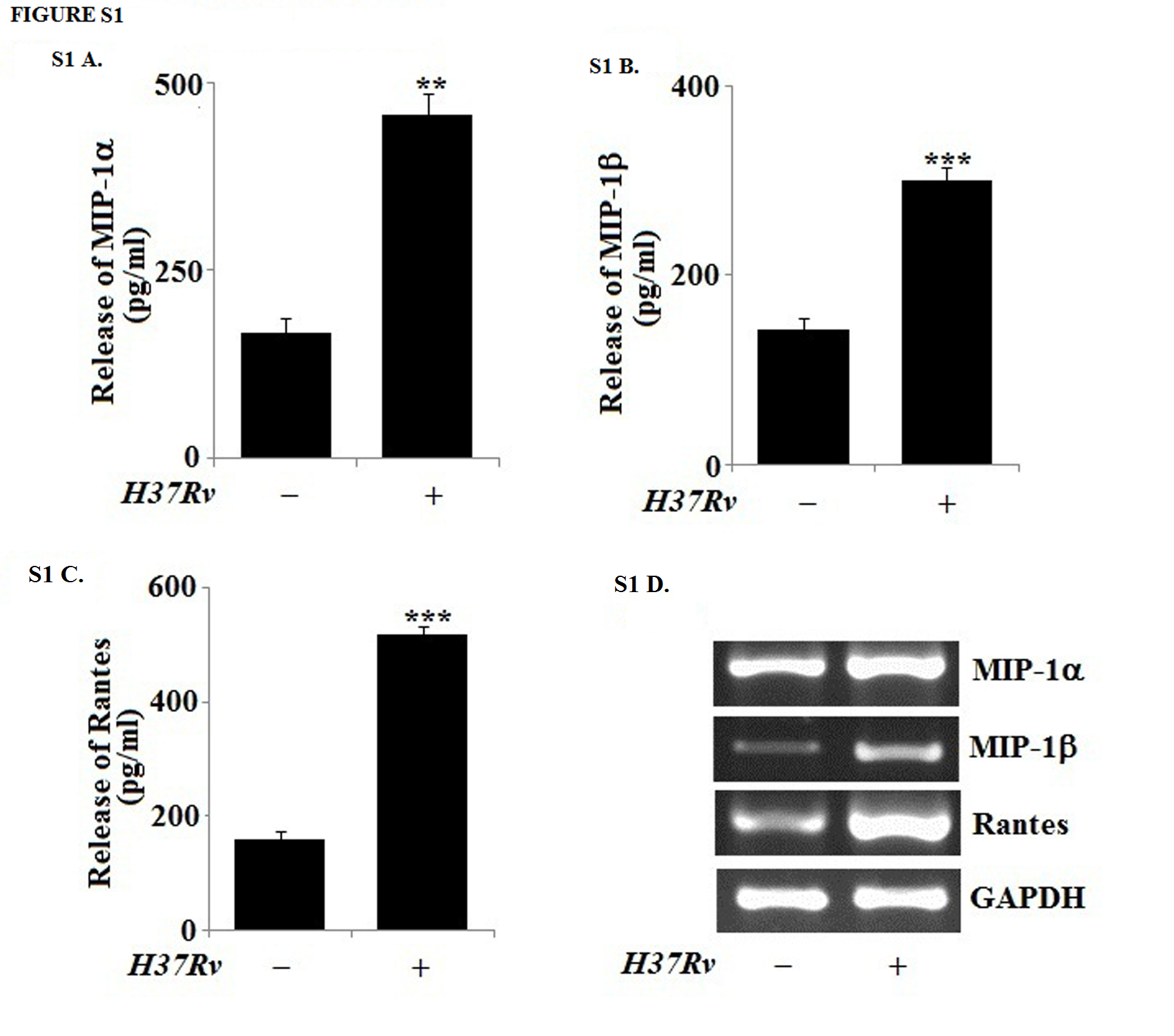

Supplement: Figure S1 — Production of chemokines during M. tuberculosis infection. Bone marrow derived macrophages (2×10^6^ cells/ml) were infected with Mycobacterium tuberculosis H37Rv (MOI = 1∶10) for 24 h and assayed for the levels of MIP-1α (A), MIP-1β (B) and Rantes (C) in the culture supernatant by ELISA as described in Methods. ELISA data are expressed as means ± standard deviations of values from triplicate experiments that yielded similar observations. ***P<.001 and **P<.05 compared to that of the uninfected control macrophages. In a separate set of experiment, macrophages were infected with Mycobacterium tuberculosis H37Rv for 3 h. The extracellular bacteria were removed from the culture plate and the macrophages were incubated for another 3 hrs. RNA was isolated and semi quantitative RT-PCR analysis for MIP-1α, MIP-1β, Rantes and GAPDH were done (D). Data represented here are from one of three independent experiments, all of which yielded similar results. (TIF) [file pone.0092477.s001.tif]

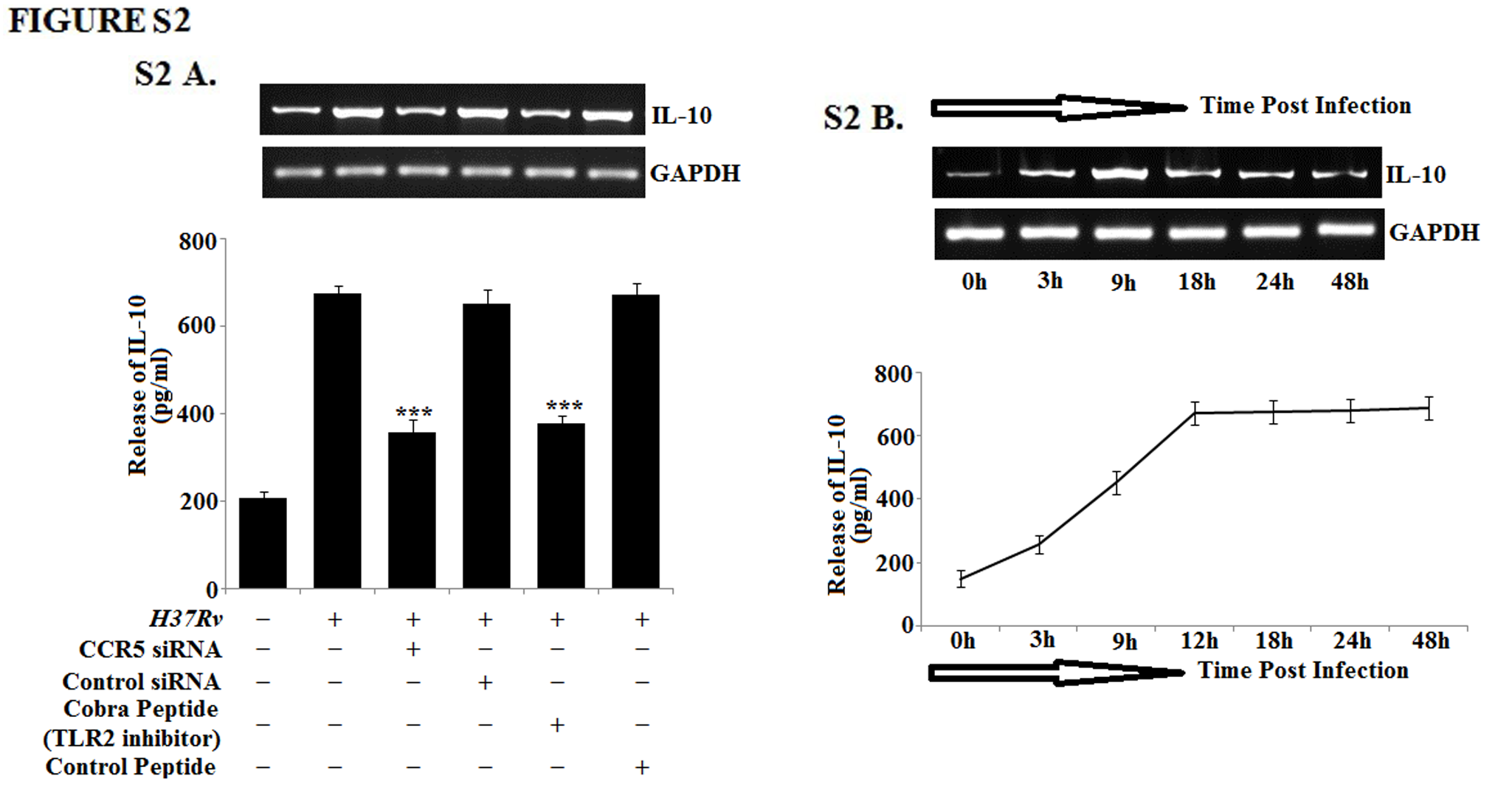

Supplement: Figure S2 — Involvement of TLR-2 and CCR5 in the M. tuberculosis elicited IL-10 production. Bone marrow derived macrophages (2×10^6^cells/ml) were treated with CCR5 siRNA, control siRNA, TLR-2 inhibitory peptide (Cobra peptide) and control peptide. The macrophages were then infected with Mycobacterium tuberculosis H37Rv (MOI = 1∶10) for 24 h and assayed for the levels of IL-10 in the culture supernatant as described above (A). ELISA data are expressed as means ± standard deviations of values from triplicate experiments that yielded similar observations. ***P<.001 and **P<.05 compared to that of the uninfected control macrophages. In a separate set of experiment, macrophages were treated as above and infected with Mycobacterium tuberculosis H37Rv for 3 h. The extracellular bacteria were removed from the culture plate and the macrophages were incubated for another 3 hrs. RNA was isolated and semi quantitative RT-PCR analyses for IL-10 and GAPDH were done. Data represented here are from one of three independent experiments, all of which yielded similar results. In a separate set of experiment, macrophages were infected with Mycobacterium tuberculosis H37Rv for different time points (B). RNA was isolated and semi quantitative RT-PCR analyses for IL-10 and GAPDH were done. Mycobacterium tuberculosis H37Rv (MOI = 1∶10) infected macrophages assayed for the levels of IL-10 in the culture supernatant as described above (A). ELISA data are expressed as means ± standard deviations of values from triplicate experiments that yielded similar observations. (TIF) [file pone.0092477.s002.tif]

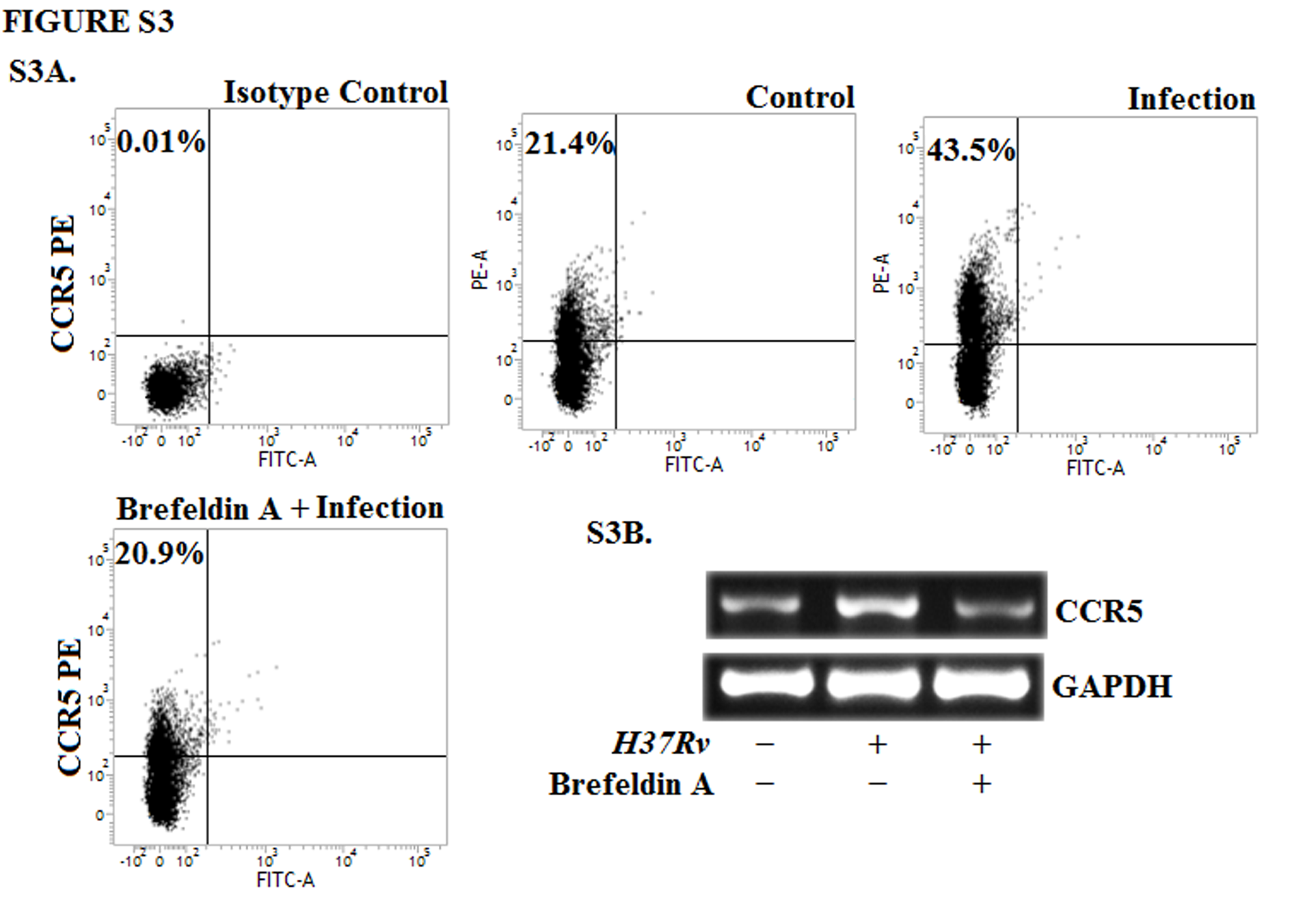

Supplement: Figure S3 — Involvement of cytokine in the M. tuberculosis elicited CCR5 expression. Bone marrow derived macrophages (2×10^6^) were cultured. The macrophages were treated with Brefeldin A and then infected with Mycobacterium tuberculosis H37Rv (MOI = 1∶10) for 24 hrs. The cell supernatants were mixed with the culture media of uninfected macrophages. Infected macrophages were analyzed by flow cytometry for CCR5 (PE) expression as described in material method (A). In a separate set, the macrophages were treated above and changes in messenger RNA (mRNA) expression of CCR5 and GAPDH were determined by semi quantitative RT-PCR (B). Data represented here are from one of three independent experiments, all of which yielded similar results. (TIF) [file pone.0092477.s003.tif]

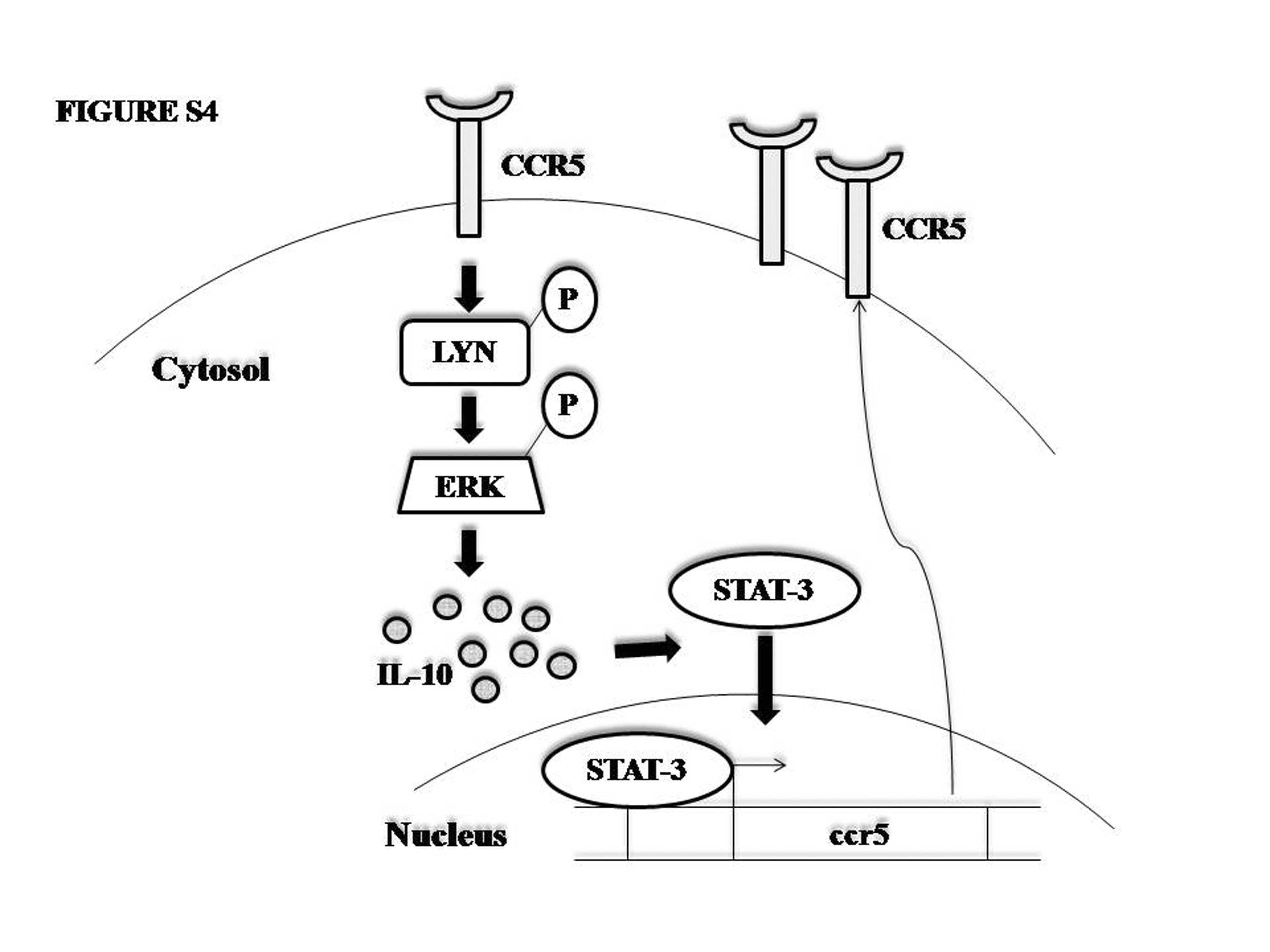

Supplement: Figure S4 — Schematic representation of the CCR5 signaling pathway during Mycobacterium infection in macrophages. During early time point of H37Rv infection, the CCR5 expression is up-regulated, which in turn activates the Lyn kinase. Phosphorylated Lyn further activates the MAP kinase ERK1/2. This signaling is culminated with the production of IL-10 in the infected macrophages. The IL-10 produced from the infected macrophages recruits STAT-3 to the CCR5 promoter of the infected macrophage and further upregulates the CCR5 expression in the infected macrophages through feedback loop mechanism. (TIF) [file pone.0092477.s004.tif]
